# A meta fusion model combining geographic data and twitter sentiment analysis for predicting accident severity

**DOI:** 10.1038/s41598-025-91484-0

**Published:** 2025-07-11

**Authors:** Areeba Naseem Khan, Yaser Ali Shah, Wasiat Khan, Amaad Khalil, Jebran Khan

**Affiliations:** 1 Department of Computer Science, COMSATS University Islamabad, Attock Campus, Attock, 43600 Pakistan; 2 Department of Software Engineering, University of Science and Technology, Bannu, Khyber Pakhtunkhwa Pakistan; 3 Department of Computer Systems Engineering, University of Engineering and Technology, Peshawar, 25000 Pakistan; 4 Department of Artificial Intelligence, Ajou University, Suwon, South Korea

**Keywords:** Computer science, Information technology

## Abstract

In recent years, advancements in deep learning and real-time data processing have significantly enhanced traffic management and accident prediction capabilities. Building on these developments, this study introduces an innovative approach ConvoseqNet to improve traffic accident prediction by integrating traditional traffic data with real-time social media insights, specifically using geographic data and Twitter sentiment analysis. ConvoseqNet combines Convolutional Neural Networks (CNNs) with Long Short-Term Memory (LSTM) networks in a sequential architecture, enabling it to effectively capture complex spatiotemporal patterns in traffic data. To further enhance prediction accuracy, a meta-model called MetaFusionNetwork is proposed, which combines predictions from ConvoseqNet and a Random Forest Classifier. Results show that ConvoseqNet alone achieved the highest predictive accuracy, demonstrating its capacity to capture diverse accident-related patterns. Additionally, MetaFusionNetwork’s performance highlights the advantages of combining model outputs for better prediction. This research contributes to real-time data-driven traffic management by leveraging innovative data fusion techniques, improving prediction accuracy, and providing insights into model interpretability and computational efficiency. By addressing the challenges of integrating heterogeneous data sources, this approach presents a significant advancement in traffic accident prediction and safety enhancement.

## Introduction

The rapid increase in traffic accidents worldwide has necessitated the development of advanced predictive systems to mitigate their occurrence and improve road safety. One promising approach is the use of deep learning techniques for real-time traffic accident prediction. Deep learning a subset of machine learning employs neural networks with multiple layers to analyze and learn from vast amounts of data. This technique has shown remarkable potential in identifying patterns and predicting outcomes based on complex datasets. The integration of deep learning into traffic management systems aims to forecast accidents before they happen enabling timely interventions that can save lives and reduce economic losses.

In recent years, advancements in deep learning and real time data processing have significantly impacted various domains including traffic management and accident prediction. The primary goal of real-time traffic monitoring systems is to enhance road safety and optimize traffic flow by employing advanced computational techniques (Priyanka 2022)^1^. A prominent example is the development of fast and deep learning algorithms that facilitate real-time traffic analysis and accident prediction. For instance, Annam et al. (2023) developed a deep learning framework that rapidly processes traffic data thereby improving response times and predictive accuracy in traffic management systems^2^.

The integration of real-time data processing in traffic systems is exemplified by the work of Ma et al. (2022) who explored energy-efficient solutions for traffic monitoring. Their research highlights the importance of real-time data in reducing energy consumption while maintaining high accuracy in traffic flow analysis^3^. Similarly, Upadhya et al. (2022) proposed a modeling approach that utilizes recent advances in machine learning to predict traffic patterns, demonstrating significant improvements in prediction accuracy over traditional methods^4^.

Innovative approaches to real-time traffic monitoring are further demonstrated by Kats et al. (2022) who introduced TraCon a substantial improvement in traffic congestion analysis. This system leverages deep learning algorithms to provide real-time insights into traffic conditions, enabling more efficient traffic management^5^. Deep learning’s application in road safety is well documented,d with research indicating its potential to significantly reduce accidents. Byoungsuk Ji (2019) developed a deep learning model that predicts road safety risks, providing actionable insights that can prevent accidents^6^. Similarly, Nicolette et al. (2020) focused on predicting accident hotspots using real-time data, demonstrating the efficacy of their model in identifying high-risk areas and informing preventative measures^7^.

In the realm of traffic accident prediction, Parsa (2019) presented a deep learning-based system that processes sensor data to predict accidents in real time. Their research underscores the potential of sensor integration in enhancing the predictive capabilities of traffic management systems^8^. Complementing this, Upadhya et al. (2022) discussed the role of real-time traffic data in reducing accident response times, emphasizing the need for efficient data processing frameworks.

Liu et al. (2017) explored real-time data integration in online traffic monitoring systems, highlighting its benefits in improving accuracy and response times. Their study demonstrates that integrating real-time data streams can significantly enhance the performance of traffic monitoring systems, making them more responsive to dynamic traffic conditions^9^. Similarly, Pillai (2021) examined the role of real-time analytics in traffic management, showing how advanced computational techniques can provide deeper insights into traffic patterns and enhance decision-making processes^10^.

Furthering the application of real-time processing, Divya V. (2024) developed a real-time traffic accident prediction model that uses machine learning algorithms to process vast amounts of traffic data. Their findings indicate that real-time processing significantly improves the accuracy of accident predictions thereby enhancing road safety^11^. This is supported by Basso (2021), who demonstrated that real-time analytics could effectively predict traffic accidents, allowing for timely interventions^6^.

The impact of real-time data processing on traffic management is also evident in the work of Ghahremannezhad (2022), who developed a system that uses real-time data to monitor and manage traffic flows. Their research highlights the potential of real-time data in optimizing traffic management systems and improving overall traffic efficiency^12^. Additionally, Zhengjing Ma (2021) examined the analytical methods for identifying vulnerable road segments emphasizing the importance of real time data in enhancing road safety and reducing accident rates^3^.

Real-time traffic monitoring systems also play a crucial role in intelligent transport systems. Changxi (2021) investigated the application of intelligent transport systems in real-time traffic management, demonstrating their effectiveness in improving traffic flow and reducing congestion. Their study highlights the importance of integrating real-time data into intelligent transport systems to enhance efficiency and reliability^13^.

The research problem addressed in this paper revolves around developing a real-time traffic accident prediction system that integrates traditional traffic accident data with real-time social media data. A key challenge in integrating these data sources is their structure, time scale, and format differences. Traditional traffic data is often structured, containing historical records and sensor data, while social media data (e.g., from Twitter) is unstructured, dynamic, and often noisy. This creates a significant challenge in terms of merging and aligning these data types effectively. To address this, the proposed ConvoseqNet model utilizes advanced data fusion techniques and preprocessing steps to harmonize these diverse data streams. The model incorporates several techniques, including Random Forest Classifier, Convolutional Neural Networks (CNN), and Long Short-Term Memory (LSTM) networks. Another proposed model is the meta-fusion model MetaFusionNetwork, which combines the strengths of multiple models. The utilization of both structured and unstructured data sources, along with advanced deep learning techniques, underscores the study’s innovative approach to traffic accident forecasting. The goal is to predict traffic accidents more accurately and earlier, which could lead to faster response times, improved traffic management, and enhanced road safety.

The key contributions of this study are as follows:

1. *ConvoseqNet Model:* A novel hybrid traffic accident prediction model that combines Convolutional Neural Networks (CNN) and Long Short-Term Memory (LSTM) architectures, enabling effective capture of complex temporal and spatial patterns in both structured and unstructured data.
2. *MetaFusionNetwork:* A unique fusion model that integrates predictions from multiple models (CNN, LSTM, XGBoost, CatBoost) to enhance classification power and generalizability, offering improved predictive accuracy across diverse datasets.
3. *Integration of Real-Time Social Media Data:* Introduction of Twitter sentiment analysis alongside traditional traffic accident datasets, significantly enhancing prediction accuracy by providing timely and relevant context to traffic accident forecasting.
4. *Comprehensive Preprocessing and Feature Engineering:* An advanced approach to preprocessing and feature engineering that merges structured traffic accident data with unstructured social media text, ensuring more robust and reliable predictions.
5. *Sentiment Analysis in Traffic Forecasting:* The novel application of sentiment analysis from social media platforms, particularly Twitter, for real-time traffic accident forecasting provides valuable insights for better traffic management and prediction.

The paper is structured as follows: The Introduction presents the motivation for using deep learning in traffic accident prediction and discusses the benefits of real-time data integration. The Related Work section reviews previous studies on traffic accident prediction, highlighting advancements in machine learning and data processing techniques. In the Methodology section, the data collection and preprocessing steps are outlined, detailing how accident data and social media insights were merged. This section also explains the hybrid modeling approach, including the use of Random Forest, the proposed ConvoseqNet, and the proposed MetaFusionNetwork models. The Experimental Setup elaborates on the specific models used, including their training and evaluation processes. The Results section compares the performance of the individual models and the proposed models with a focus on accuracy and predictive reliability. Finally, the Discussion and Conclusion sections summarize the findings and implications emphasizing the improvements offered by the ensemble approach and suggesting avenues for future research.

## Related work

The emergence of autonomous vehicles has also benefited from advancements in real-time data processing and deep learning. Autonomous driving relies heavily on the real-time analysis of vast amounts of data from various sensors and cameras to make split-second decisions. For example, Wang et al. (2018) discussed how deep learning algorithms are integral to the perception and decision-making processes in autonomous vehicles improving their ability to navigate complex traffic environments safely and efficiently^9^.

Furthermore, the use of real-time data in smart city initiatives has become increasingly prominent. Smart cities aim to leverage technology to enhance urban living and real-time traffic monitoring is a key component of these efforts. P. U. Anitha. (2024) explored how integrating real-time traffic data with other smart city technologies can lead to more sustainable and efficient urban mobility solutions. This integration not only improves traffic management but also reduces the environmental impact of urban transportation systems^14^.

In addition to improving traffic management and accident prediction, real-time data processing has applications in public transportation systems. Efficient public transportation is critical for reducing urban congestion and pollution. Lee et al. (2020) developed a real-time monitoring system for public buses that uses deep learning to predict delays and optimize routes enhancing the reliability and efficiency of public transport services^15^.

The advancements in deep learning and real-time processing also hold promise for emergency response systems. Rapid and accurate traffic accident prediction and monitoring enable quicker emergency response times potentially saving lives. Chen et al. (2021) developed a real-time emergency response system that integrates traffic data to provide first responders with optimal routes and accurate accident location information significantly improving the efficiency of emergency services^16^.

The rapid increase in traffic accidents worldwide has necessitated the development of advanced predictive systems to mitigate their occurrence and improve road safety. One promising approach is the use of deep learning techniques for real-time traffic accident prediction. Deep learning a subset of machine learning employs neural networks with multiple layers to analyze and learn from vast amounts of data. This technique has shown remarkable potential in identifying patterns and predicting outcomes based on complex datasets. The integration of deep learning into traffic management systems aims to forecast accidents before they happen enabling timely interventions that can save lives and reduce economic losses.

Further extending this research Cai et al. (2020) applied deep generative models for real-time crash prediction on expressways. Their study highlighted the ability of deep learning methods to capture the intricate relationships between various traffic parameters and accident occurrences significantly improving the accuracy of crash predictions compared to traditional statistical methods^15^. Azhar et al. (2023) focused on the detection and prediction of traffic accidents using deep learning techniques. Their model was designed to process spatiotemporal data effectively identifying and predicting traffic collisions in real time. This approach demonstrates the versatility of deep learning in handling different types of data for comprehensive accident prediction^17^.

Another significant contribution to this field is the work by Lin et al. (2020), who developed an intelligent traffic accident prediction model for the Internet of Vehicles (IoV). By leveraging deep learning techniques their model was able to analyze real-time traffic conditions and predict potential accident risks facilitating proactive measures to prevent collisions^18^. Shuai Liu. (2020) applied deep learning techniques to predict traffic accidents using spatiotemporal sequential data. They utilized Long Short-Term Memory (LSTM) and Gated Recurrent Unit (GRU) networks to process real-time traffic data achieving high accuracy in predicting traffic incidents^19^.

The study by Babbar. (2023) on citywide traffic accident risk prediction further emphasized the potential of deep learning in managing urban traffic systems. Their model utilized big traffic data and deep learning to predict traffic flow and accidents highlighting the scalability of these techniques for large-scale implementations^20^. Simi Asher (2020) explored real-time traffic accidents post-impact prediction using crowdsourcing data. Their study utilized machine learning methods to analyze continuously updated traffic conditions enabling real-time assessment and prediction of traffic accident durations^21^.

Formosa et al. (2020) introduced a deep learning model for predicting real-time traffic conflicts integrating large volumes of heterogeneous data to enhance the accuracy of their predictions. Their centralized digital architecture effectively managed and processed diverse traffic data sources and Basso et al. (2021) developed a real-time crash prediction model using vehicle-by-vehicle data marking the first operational real-time accident prediction software. Their deep learning-based approach demonstrated significant improvements in predicting highway segment accidents in real time. Finally, Li et al. (2022) proposed a hybrid deep learning model for real-time traffic incident detection. Their model combined various deep learning techniques to achieve immediate detection of traffic incidents further proving the efficacy of deep learning in real-time traffic management^22^.

Recent advancements in machine learning (ML) have contributed significantly to the development of systems for predicting traffic accident severity and preventing accidents. Despite the extensive efforts made by the automotive industry to enhance vehicle safety traffic accidents continue to occur and understanding the causes of these accidents remains crucial. Various machine learning algorithms such as decision trees, Random Forest, support vector machines (SVM), and ensemble methods have been applied to predict accident severity and improve road safety^23^. Studies have shown that machine learning techniques like logistic regression, decision trees, deep neural networks, and geospatial analysis are particularly useful in predicting accident hotspots and improving traffic flow^24^. Furthermore, recent research has examined accident prediction as a classification problem focusing on predicting accident occurrence and severity by analyzing contributing factors such as weather conditions, road type, and traffic volume^25^. Additionally, in the maritime industry predictive models like ARIMAX have been applied to forecast accidents demonstrating the potential of ML models to predict accidents in various sectors^26^. With ongoing research, the integration of advanced optimization algorithms and real-time data, including social media insights is expected to further enhance predictive accuracy and reduce accident-related fatalities^27^.

Several studies have focused on predicting traffic accident severity using machine learning (ML) and data analysis techniques offering innovative approaches to enhance prediction accuracy and safety measures. For marine accidents, a two-stage feature selection method was developed to select and rank Risk Influential Factors (RIFs) significantly improving the accuracy of severity predictions using ML models like LightGBM. The study also analyzed risk control measures from a quantitative perspective contributing to AI-driven safety assessments^28^. Similarly, traffic accident severity in the Chinese National Automobile Accident In-Depth Investigation System was predicted by including innovative features such as accident location and collision speed with Random Forest ranking the importance of factors to optimize prediction models^29^. In a different study ML techniques were applied to large-scale road accident data, using classification methods like decision trees and random forests to predict accidents and understand influencing factors offering strategies for accident prevention^30^. Additionally, a tool was developed to predict traffic accident risks in Portugal revealing that time of day and weather conditions like rain had a significant impact on accident probabilities^31^. Lastly, a multi-graph learning framework called MG-TAR was proposed to predict accident risk by modeling spatio-temporal relationships between dangerous driving and accidents reducing prediction errors and improving accuracy in identifying high-risk areas^32^.

Several studies have focused on improving road safety by predicting various factors contributing to traffic accidents, including driver behavior, drowsiness, and accident severity. A novel Driver Decision Support System (DDSS) was developed to predict abnormal driving behaviors using K-Means clustering and compare its efficacy with other algorithms like SVM and Decision Trees. This system aims to prevent accidents by advising nearby vehicles to change lanes or alter speed based on predicted behaviors^33^. In addition, driver drowsiness, a major cause of accidents, has been extensively studied. A review of various detection methods highlights the importance of using physiological, vehicle-based, subjective, and behavioral measures to warn drivers of impending accidents^34^. The integration of deep learning techniques in traffic accident prediction is also explored, with a bi-directional ConvLSTM U-Net model proposed for predicting accidents in specific road grids. The model showed superior accuracy compared to traditional methods in predicting motor vehicle, non-motor vehicle, and single-vehicle accidents^35^. Social media data especially Twitter has also been incorporated into accident prediction models. One such study combined tweet data with sentiment analysis, weather conditions, and geospatial information to predict road accidents using deep learning techniques^17^. Finally, Artificial Neural Networks (ANNs) were used to predict traffic accidents and their severity in Serbia and Bosnia, demonstrating good generalization capabilities in predicting accidents based on road and traffic-related factors^36^. Road traffic accidents (RTAs) are a leading cause of death especially in low and middle-income countries like Rwanda. This study uses Random Forest (RF) and Support Vector Machine (SVM) models to forecast short-term road accidents with RF outperforming SVM in terms of prediction accuracy. Machine learning models show great potential in enhancing road safety by guiding policymakers and healthcare providers in accident prevention efforts^37^. Another study introduces AccidentGPT a multi-modal large model for accident analysis and prevention offering advanced traffic safety capabilities for autonomous and human-driven vehicles as well as real-time traffic management^38^.

As urban traffic congestion rises, predicting accidents has become crucial for city planning and public safety. This study compares modern deep learning models, like the Transformer, with traditional time series models (ARIMA, Prophet) to forecast accidents using feature importance analysis and introducing real-time interventions with large language models (LLMs) like LLaMA-2. It highlights the integration of multimodal models in enhancing autonomous driving systems and urban safety^39^. Another study investigates machine learning algorithms (Decision Tree, LightGBM, XGBoost) for predicting road traffic accident severity in the UK emphasizing the importance of vehicle inspection and traffic policy for reducing accident severity^40^. A third study applies Support Vector Machine (SVM) models to predict aircraft accident severity introducing new factors based on inattentional blindness theory and evaluating model accuracy using cross-validation and confusion matrices^41^.

Another study addresses the challenges in predicting nuclear power plant (NPP) accidents by proposing an algorithm that predicts long-term plant behavior while providing uncertainty information^42^. Using bidirectional LSTM and attention mechanisms along with a variational autoencoder the algorithm forecasts multivariate plant parameters for 2 hours showing high accuracy in simulations for a Westinghouse NPP^43^. Similarly, a multi-task learning framework (TAP) using Spatio-temporal Variational Graph Auto-Encoders (ST-VGAE) captures dynamic correlations in traffic data for accident prediction with experiments showing superior performance^44^. Another work presents a VRU collision prediction system using LSTMs on V2X communication data and a study on traffic crash prediction uses AI to improve safety management with real-time monitoring of traffic dynamics^45^. Finally, a dynamic multi-graph neural network (DMGNN) is proposed to enhance traffic flow prediction by incorporating accident-related disruptions and dynamically adjusting the graph structure for more accurate predictions^46^.

These studies collectively highlight the transformative potential of deep learning in real-time traffic accident prediction. By leveraging advanced neural network architectures and integrating real-time data these models can significantly enhance the accuracy and timeliness of traffic accident predictions paving the way for safer and more efficient transportation systems. As technology progresses, the potential for even more sophisticated and efficient traffic management solutions will likely expand further enhancing the safety and efficiency of transportation systems worldwide.

## Methodology

The methodology section outlines the approach and techniques employed to develop a robust traffic accident prediction system by integrating both traditional accident data and real-time social media insights.

### Data description

The traffic accident prediction system integrates two distinct data sources: traditional accident data and real-time social media data from Twitter. The accident dataset, sourced from various government entities and traffic monitoring systems, covers traffic incidents across 49 US states from February 2016 to March 2023, comprising approximately 7.7 million records. The dataset includes crucial features such as location, time, weather conditions, and accident severity, all of which are vital for accurate prediction. To ensure data integrity, this dataset undergoes rigorous preprocessing, including cleaning to remove missing values and conversion of time-related information into a standardized date-time format.

On the other hand, the social media dataset is derived from Twitter using the Twitter API, specifically targeting tweets related to traffic incidents. Keywords like ”car accident” and ”traffic collision” are used to identify relevant tweets. This dataset, being unstructured, requires substantial preprocessing, which involves tokenization, stop-word removal, and filtering of non-alphabetic tokens. The processed text is then analyzed through natural language processing (NLP) methods to extract meaningful information such as accident locations, time references, and mentions of accident severity.

**Fig. 1:**
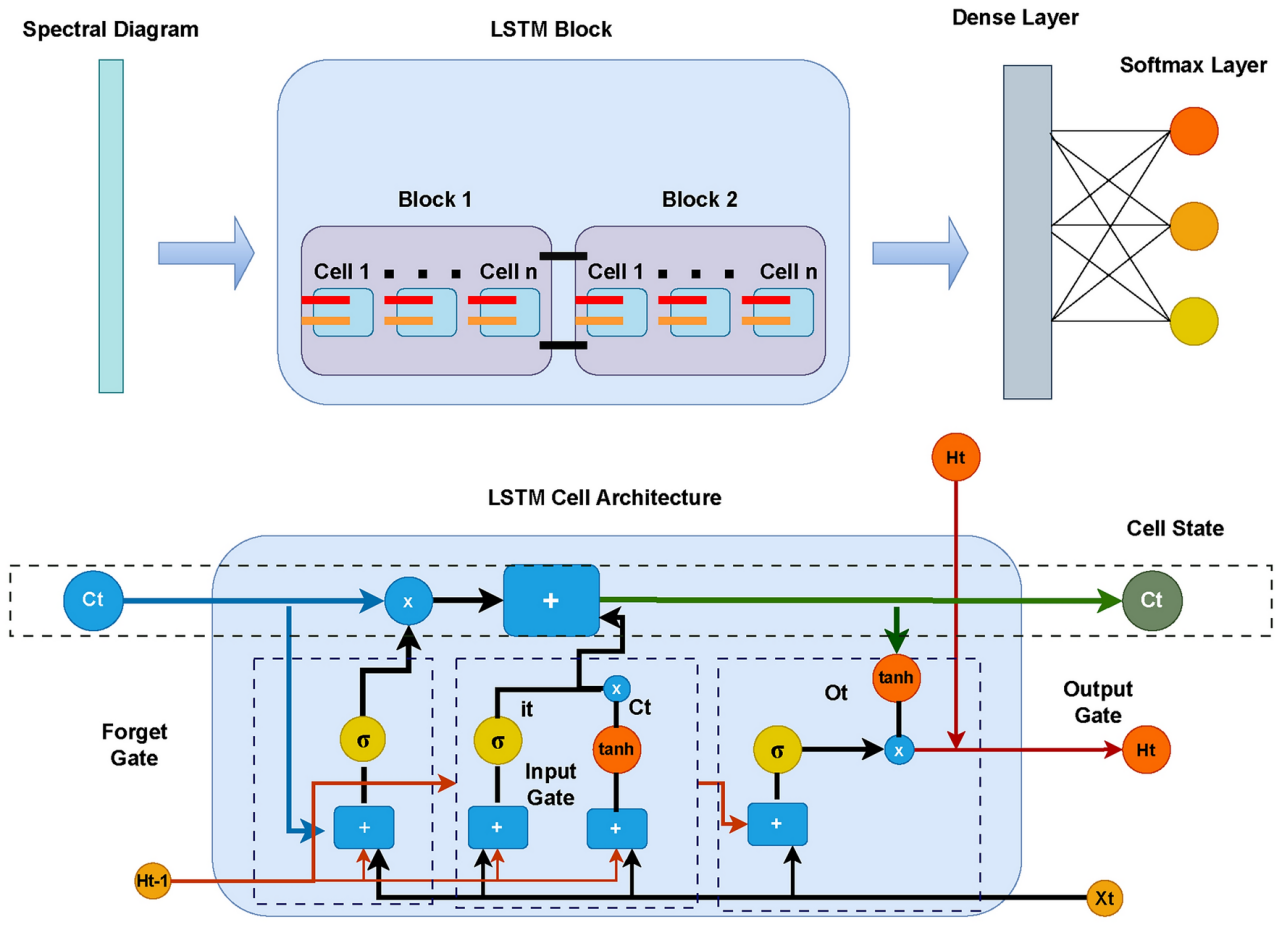
Traditional workflow of LSTM.

### Data quality and bias

Given the disparate nature of the datasets one structured and the other unstructured ensuring high quality data integration is critical for building a reliable predictive model. For the accident dataset quality control measures focus on verifying the accuracy of geographic data ensuring consistency in timestamps and removing irrelevant or out-of-scope records. For the Twitter dataset quality control includes filtering out irrelevant tweets, such as those not related to traffic accidents and ensuring geographic information in tweets is accurately mapped to real-world locations. This is crucial as tweets often contain noisy or incomplete location data which could lead to mismatches during the data merging process.

One of the significant challenges in using Twitter data is the potential for biases. These biases may stem from various factors such as sample biases (e.g., only certain geographic areas or types of accidents being more frequently tweeted about) or noise in the data (e.g., irrelevant tweets or sarcastic remarks). Additionally, the volume of social media posts can introduce significant fluctuations in data quality due to the unstructured nature of user-generated content. Such biases can negatively impact the model’s performance leading to skewed predictions. For instance, if certain accident types or locations are overrepresented in Twitter data the model might place undue emphasis on these areas reducing its generalizability. Similarly, noise in the social media data like irrelevant posts or sentiment that does not correlate with actual accident severity could degrade the model’s accuracy.

To mitigate these biases several strategies are employed:

1. Geographical Filtering: Tweets are only considered if their location data matches accident records within a specified radius reducing location mismatches.
2. Text Preprocessing: Aggressive filtering is applied to remove irrelevant content such as spam or general traffic discussions, ensuring that only pertinent accident-related information is included.
3. Sentiment Analysis Adjustments: Sentiment analysis models are fine-tuned to filter out tweets with excessive sarcasm or exaggeration thus enhancing the quality of sentiment-related features.
4. Bias Detection and Adjustment: Analyzing the distribution of tweets across different regions and accident types and adjusting the model to account for overrepresented or underrepresented categories.

By addressing these biases through careful data preprocessing and model adjustments, the accuracy of the traffic accident prediction system is improved, enhancing its ability to provide reliable, real-time insights for accident prevention.

### Feature selection and fusion strategies

In this study, we employ advanced feature selection and fusion strategies to enhance the predictive performance of the traffic accident severity model. Feature selection ensures that only the most relevant and impactful features from both structured accident data and unstructured social media data are used in training. For the structured accident data we apply correlation analysis, Recursive Feature Elimination (RFE) and Random Forest feature importance to identify the most predictive features, such as location, weather conditions and time of day. These features are then retained for the model while redundant or irrelevant features are discarded to avoid overfitting and ensure efficient computation.

For the unstructured Twitter data the raw text is processed using CountVectorizer and TF-IDF techniques to transform it into a numerical format suitable for machine learning models. We also explore the use of word embeddings like Word2Vec and GloVe which capture semantic meaning and relationships between words. This helps the model better understand contextual insights from the tweets related to accidents. Additionally, stop-word removal and token filtering are applied to eliminate noise and focus on meaningful words that could influence the prediction of accident severity.

After feature selection, we explore two main fusion strategies. Early Fusion involves combining the processed structured and unstructured features into a single input dataset, which is then fed into the machine learning model. To ensure that the numerical and text features are on the same scale, we apply MinMaxScaler for normalization. This fusion strategy allows the model to learn directly from the integrated data.

In contrast, Late Fusion involves training separate models for the structured and unstructured datasets. The outputs of these models are then combined through voting mechanisms (hard or soft voting) to make the final prediction. This strategy allows for specialized learning of the individual datasets before their predictions are merged.

Finally, we explore a hybrid fusion approach the MetaFusionNetwork where predictions from different models such as ConvoseqNet, XGBoost and CatBoost are combined and passed through a logistic regression model to generate the final prediction. This method leverages the strengths of both deep learning (for spatial and temporal feature extraction) and gradient boosting models (for structured data) to improve prediction accuracy and robustness.

### Model development and training

For model development, a classification model is trained to predict accident severity using features from both datasets. Features include geographical coordinates and the sentiment of tweets. The text data is converted into numerical features using CountVectorizer which transforms the cleaned text into a matrix of token counts. These text features are then combined with numerical features from the accident dataset. The combined dataset is split into training and validation sets to ensure robust model evaluation.

A Random Forest Classifier model is trained using the training data, with its results later merged in the metafusion model. This model is chosen for its ability to handle large datasets and its effectiveness in dealing with both numerical and categorical data. The model’s performance is evaluated on the validation set using metrics such as accuracy, precision, recall and F1-score. These metrics provide a comprehensive understanding of the model’s performance highlighting areas where it excels and where it needs improvement. Evaluation and optimization of the model involve continuous monitoring of its performance and incorporating additional features to enhance accuracy. Hyperparameter tuning is conducted to find the optimal settings for the model improving its predictive power. The inclusion of supplementary data such as weather conditions and traffic flow information can further refine the model’s predictions making it more reliable and comprehensive.

The potential impact of this project is substantial. Faster and more accurate identification of severe accidents can significantly improve emergency response times ensuring that resources are deployed where they are needed most. The insights gained from the model can inform urban planning and road safety measures leading to safer and more efficient transportation systems. Additionally, real-time dissemination of traffic information can raise public awareness about current traffic conditions promoting safer driving behaviors and reducing accident rates. This innovative approach leverages the power of social media data, which is often underutilized in traffic management systems. By combining traditional traffic data with real-time social media insights the system provides a more accurate and timely prediction of traffic accident severity. This methodology showcases the potential of integrating diverse data sources to enhance the capabilities of traffic management systems paving the way for smarter and safer roads. In summary, the project encompasses several critical steps from data collection and preprocessing to feature engineering, model development and real-time implementation. Each step is designed to maximize the utility of the available data ensuring that the predictive model is both accurate and efficient. The deployment of the model in a real-time setting highlights its practical applicability demonstrating its potential to make a significant impact on road safety and traffic management.

By addressing the challenges of modern traffic management through innovative use of data and technology, this project offers a valuable contribution to the field. The methodology detailed here provides a roadmap for similar initiatives emphasizing the importance of data integration and real-time processing in developing effective traffic management solutions. The continuous evaluation and optimization of the model ensure that it remains relevant and accurate, adapting to changing traffic patterns and conditions.

Overall, this approach exemplifies how advanced computational techniques can be applied to real-world problems, offering a practical solution that enhances safety and efficiency on the roads. The integration of accident and social media data represents a significant step forward in traffic management, providing a more comprehensive understanding of traffic dynamics and enabling proactive measures to prevent accidents and improve traffic flow.

**Training Phase**

1. Bootstrap Sampling:

- For each tree $n = 1, 2, ..., N$:
  - Sample $N$ data points with replacement from the training dataset. This forms a bootstrap sample.

2. Feature Selection:

- For each tree:
  - Randomly select a subset of features $m$ out of $M$ total features. The value of $m$ is usually much less than $M$, typically $\sqrt{M}$ for classification.

3. Tree Growing:

- Grow a decision tree using the selected features and the bootstrap sample:
  - At each node of the tree:
    * Randomly select $m$ features.
    * Choose the best feature/split among the $m$ features using a criterion such as Gini impurity or entropy.
    * Split the node based on the selected feature/split.
    * Repeat until a stopping criterion is met (e.g., maximum depth reached, minimum samples per leaf).

**Prediction Phase:**

For a new input sample $\textbf{x}$:

- For each tree $n = 1, 2, ..., N$:
  - Traverse the tree and predict the class label based on the majority class of the leaf node reached by $\textbf{x}$.

- Aggregate the predictions from all trees:
  - For classification, use majority voting among the predictions from all trees to determine the final class label.
  - For regression, use the average of the predictions from all trees as the final output.

**Model Representation:**

Each tree $n$ in the forest can be represented as a set of decision rules $\{R_n\}$, where $R_n$ is a set of decision nodes and leaf nodes. The Random Forest Classifier combines the predictions of all trees to make the final classification decision.

**Mathematical Notation:**

$$\begin{aligned}&\textbf{x}: \text {Input feature vector.} \\&N: \text {Number of trees in the forest.} \\&M: \text {Total number of features.} \\&m: \text {Number of features considered at each split.} \\&K: \text {Number of classes (for classification tasks).} \\&R_n: \text {Set of decision nodes and leaf nodes for tree n .} \\&p(\textbf{x}): \text {Predicted class probability for input \textbf{x} .} \\&\hat{y}: \text {Predicted class label for input \textbf{x} .} \end{aligned}$$

The Random Forest Classifier can be mathematically expressed as:

$$\begin{aligned} p(\textbf{x}) = \frac{1}{N} \sum _{n=1}^{N} p_n(\textbf{x}) \end{aligned}$$

Where $p_n(\textbf{x})$ is the predicted class probability for input $\textbf{x}$ by tree $n$.

$$\begin{aligned} \hat{y} = \text {argmax}_k \left( p(\textbf{x})_k \right) \end{aligned}$$

Where $\hat{y}$ is the predicted class label for input $\textbf{x}$, and $p(\textbf{x})_k$ is the predicted probability of class $k$.

This above mathematical model illustrates how information flows through a random forest classifier.

**Fig. 2:**
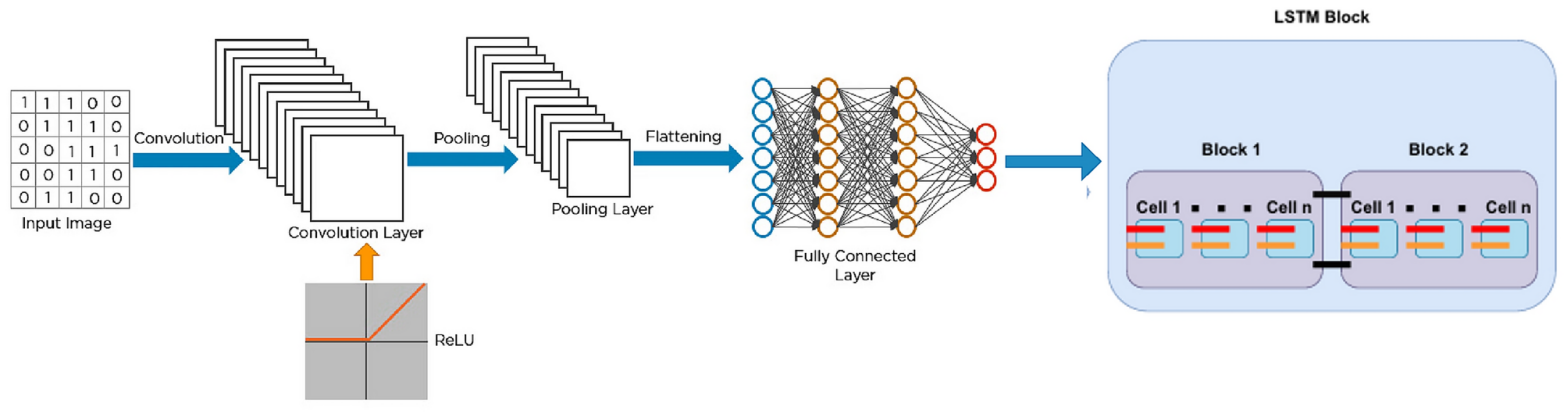
Workflow of ConvoseqNet.

To develop an advanced real-time traffic accident prediction model leveraging deep learning techniques we employed a novel convolutional neural network (ConvoseqNet) architecture that integrates both Convolutional Neural Networks (CNN) and Long Short-Term Memory (LSTM) networks for enhanced feature extraction and temporal sequence modeling. Unlike traditional models, our approach combines spatial feature extraction through Conv1D layers with bidirectional LSTMs for capturing temporal dependencies allowing for more accurate prediction of traffic accident severity. This architecture implemented using TensorFlow and Keras is specifically designed to handle sequential traffic data making it highly suitable for real-time applications.

Our ConvoseqNet model starts with an Embedding layer to transform input sequences into dense vectors. This is followed by multiple Conv1D layers with 256 filters and a kernel size of 5 which focus on learning the spatial hierarchies in the traffic data. The model then incorporates a bidirectional LSTM layer with 128 and 64 units to process the temporal dynamics of the traffic sequences. Spatial Dropout is applied to prevent overfitting and multiple Dense layers with varying neuron counts further refine the learned features.

We optimized the model with advanced hyperparameters such as using a learning rate scheduler for the Adam optimizer, a batch size of 64, and training for 30 epochs. Notably, our model employs early stopping with a patience of some epochs to avoid overfitting and checkpoints are saved based on the best validation accuracy. This methodology leveraging a unique combination of CNN and LSTM layers ensures robust real-time traffic accident prediction while maintaining high accuracy and generalizability. The overall working of LSTM and Convoseqnet is shown in Figs. 1 and 2.

**Table 1:** Hyperparameters of the advanced ConvoseqNet model.

Model	Hyperparameter	Value
ConvoseqNet	Vocabulary size	10,000
	Embedding dimension	100
	Max sequence length	100
	Conv1D filters	256
	Kernel size	5
	LSTM units	128, 64
	Spatial dropout rate	0.5
	Dense layer dropout rates	0.5, 0.3
	Dense layer units	128, 64
	Batch size	64
	Epochs	30
	Optimizer	Adam
	Loss function	Sparse categorical cross-entropy
	Early stopping patience	3
	Model checkpoint	Save best only

To develop a robust traffic accident prediction model we utilize a hybrid technique that leverages the unique strengths of two proposed models ConvoseqNet and MetaFusionNetwork model rather than a traditional ensemble approach. ConvoseqNet is designed to capture complex spatiotemporal patterns in accident data by combining Convolutional Neural Networks (CNN) with Long Short-Term Memory (LSTM) networks making it well-suited for sequential and spatial feature extraction. MetaFusionNetwork builds upon ConvoseqNet’s insights by integrating it with Gradient Boosting achieving a synergy between deep learning’s representation power and Gradient Boosting’s predictive accuracy.

The process begins with data preparation which is critical for accurate model input. This phase involves processing text data where tokenization converts text into numerical sequences and padding ensures uniform sequence length essential for feeding data into the neural network. After this step, the text data is combined with numerical features to form a comprehensive dataset. Finally, the dataset is split into training and validation sets facilitating performance evaluation and optimization during training.

Once the data is prepared we move to the model training stage where three distinct models are employed to capture different aspects of the data. The first model is a neural network that is designed to learn complex patterns from the data. This network typically includes layers such as embeddings to handle text data convolutional layers to extract features and recurrent layers like LSTM or GRU to capture sequential dependencies. The neural network excels in understanding intricate patterns and relationships within the text data.

In addition to ConvoseqNet, our proposed model we also train two specialized models XGBoost and CatBoost. XGBoost utilizes gradient boosting to build a sequential ensemble of decision trees with each tree focusing on correcting errors from previous iterations to enhance predictive accuracy. This method is particularly effective for structured tabular data capturing intricate feature interactions. CatBoost another gradient boosting model is optimized for handling categorical features and constructs decision trees with enhancements that manage categorical data efficiently and help prevent overfitting making it robust across diverse data types.

Once these individual models are trained their predictions are combined to maximize their unique strengths. The combined predictions serve as a comprehensive feature set which is then passed to our proposed MetaFusionNetwork model for the final prediction. This MetaFusionNetwork model built upon logistic regression integrates the outputs of ConvoseqNet, XGBoost and CatBoost synthesizing insights across these models to enhance predictive accuracy. By leveraging the unique capabilities of each, the MetaFusionNetwork model achieves balanced and robust predictive performance across the dataset.

**Fig. 3:**
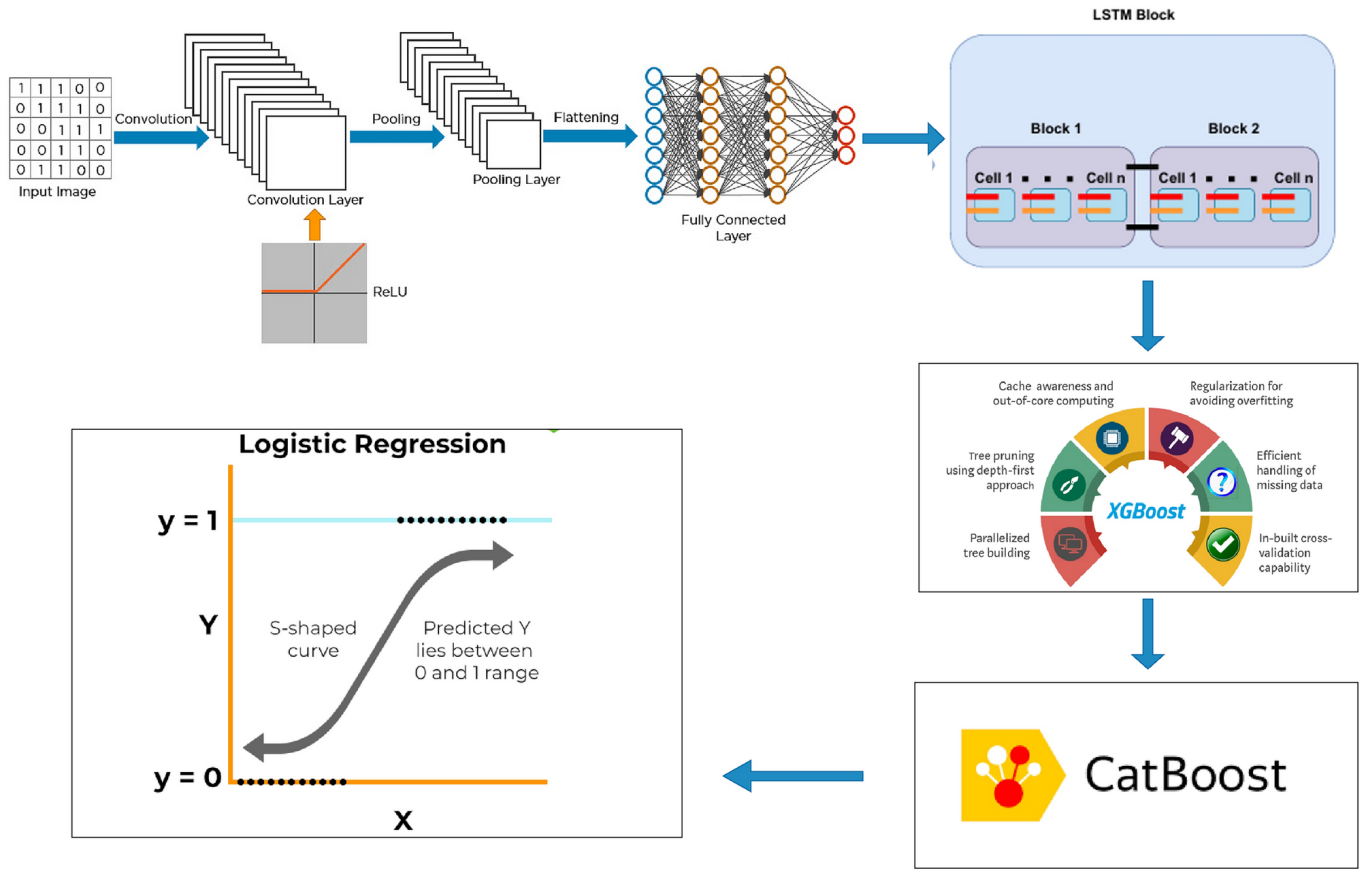
Workflow of MetaFusionNetwork.

**Table 2:** Hyperparameters table for MetaFusionNetwork.

Model	Hyperparameter	Value
ConvoseqNet	Vocabulary size	10,000
	Embedding dimension	100
	Max sequence length	100
	Conv1D filters	256
	Kernel size	5
	LSTM units	128, 64
	Spatial dropout rate	0.5
	Dense layer dropout rates	0.5, 0.3
	Dense layer units	128, 64
	Batch size	64
	Epochs	30
	Optimizer	Adam
	Loss function	Sparse Categorical Cross-Entropy
	Early stopping patience	3
	Model checkpoint	Save Best Only
Random forest	n_estimators	Default (100)
	max_depth	None (default)
	criterion	Gini
XGBoost	n_estimators	150
	learning_rate	0.1
	max_depth	10
CatBoost	iterations	150
	depth	10
	learning_rate	0.1
Logistic regression	max_iter	1000
	random_state	42

The performance of the proposed MetaFusionNetwork model is evaluated using various metrics, including accuracy, precision, recall, and F1-score on the validation set. This evaluation allows us to assess the model’s effectiveness thoroughly and provides insights into its ability to generalize to unseen data. The results from this evaluation highlight the model’s strengths and inform areas for potential refinement guiding further enhancements. In summary, this hybrid approach systematically combines deep learning and gradient boosting methods to create a robust and accurate traffic accident prediction model, specifically leveraging the unique strengths of our proposed ConvoseqNet and MetaFusionNetwork models. The mathematical formulation of this novel technique is detailed below, and workflow is explained in Figs. 2 and 3

To build this traffic accident prediction model, we apply a hybrid technique involving ConvoseqNet, XGBoost, CatBoost, and the MetaFusionNetwork model. Each component is designed to capture distinct aspects of the data, allowing the MetaFusionNetwork model to synthesize these insights into a comprehensive prediction.

To build a robust traffic accident prediction model, we propose a novel hybrid technique that integrates deep learning and gradient boosting models, specifically our own ConvoseqNet architecture and XGBoost. This approach combines ConvoseqNet’s ability to capture complex patterns in text and spatial data with the predictive power of gradient boosting on tabular data.

### ConvoseqNet model

ConvoseqNet is a hybrid deep learning model designed to handle sequential data, particularly for tasks like sentiment analysis. It leverages the combined power of Convolutional Neural Networks (CNN) and Long Short-Term Memory (LSTM) networks making it well suited to capture both spatial and temporal dependencies in text data. Initially, the model uses an embedding layer to convert text into dense vectors leveraging pre-trained embeddings to capture semantic meaning. The first component of ConvoseqNet is a convolutional layer (CNN) that processes the input sequence extracting key spatial features from the text. This is followed by max pooling and spatial dropout layers to reduce overfitting and improve generalization. These CNN layers are highly effective in identifying local patterns such as n-grams or word pairs that are crucial for understanding the context of text.

After extracting spatial features, ConvoseqNet integrates an LSTM layer to capture the sequential temporal dependencies between words. This bidirectional LSTM processes the input data both forwards and backwards enabling the model to understand context from both directions. The output of the LSTM layer is then passed through batch normalization and dense layers which further refine the features and prevent overfitting. Finally, the model produces class probabilities through a softmax layer providing the sentiment classification output. ConvoseqNet’s architecture combines the advantages of both CNN and LSTM excelling at modeling complex sequential patterns in text while also maintaining high interpretability and accuracy making it particularly suitable for tasks where understanding nuanced language patterns is critical. The ConvoseqNet model leverages convolutional and recurrent layers to effectively extract spatiotemporal patterns. Its architecture as defined in the code and also shown in Table 1 includes:

- **Embedding Layer:** Each word $w_i$ in the input sequence is converted to a dense vector $e_i$ using a pre-trained embedding matrix:
  $$\begin{aligned} e_i = W_{\text {embed}}[w_i] \end{aligned}$$
  This layer helps to represent words in a high-dimensional space, capturing their semantic meaning.
- **Convolutional Layers:** Two Conv1D layers with 512 filters each apply convolutional operations across word vectors to capture local dependencies:
  $$\begin{aligned} c_k = \text {ReLU}(W_k *e_i + b_k) \end{aligned}$$
  where $W_k$ represents convolutional filters and $b_k$ is the bias term. Batch normalization is applied after each convolution to stabilize training.
- **Max-Pooling and Spatial Dropout:** A max-pooling layer captures the most relevant features by reducing dimensionality, followed by spatial dropout to prevent overfitting by dropping entire feature maps randomly.
- **Bidirectional LSTM Layers:** Stacked Bidirectional LSTMs with 256 and 128 units are used to capture sequential dependencies from both directions:
  $$\begin{aligned} h_t = o_t \odot \tanh (c_t) \end{aligned}$$
  where $h_t$ represents the hidden state. These layers help in capturing long-range dependencies in the sequence data.
- **Dense Layers and Output:** A dense layer with 256 units, followed by a dropout layer, provides an intermediate representation. Finally, a dense layer with a softmax activation outputs the class probabilities:
  $$\begin{aligned} \hat{y} = \text {Softmax}(W_{\text {dense}} h_T + b_{\text {dense}}) \end{aligned}$$

### XGBoost model

The XGBoost component complements the deep learning model by applying gradient-boosted decision trees, specifically optimized for tabular data. The prediction is given by:

$$\begin{aligned} \hat{y}_i = \sum _{k=1}^K \alpha _k \cdot f_k(x_i) \end{aligned}$$

where:

- $K$ is the number of trees.
- $\alpha _k$ is the weight of the $k$-th tree.
- $f_k(x_i)$ is the prediction of the $k$-th tree for input $x_i$.

By combining ConvoseqNet for spatiotemporal data and XGBoost for tabular features, the model integrates deep learning’s sequential capabilities with gradient boosting’s effectiveness on structured data. This hybrid configuration is designed to provide a comprehensive and accurate approach to traffic accident prediction.

### CatBoost model

CatBoost is a gradient-boosted decision tree model optimized for categorical features:

$$\begin{aligned} \hat{y}_i = \sum _{k=1}^K \alpha _k \cdot f_k(x_i) \end{aligned}$$

where:

- Similar to XGBoost, $K$ is the number of trees.
- $\alpha _k$ is the weight.
- $f_k(x_i)$ is the prediction of the $k$-th tree.

### Logistic regression proposed meta-fusion model

The logistic regression model combines the predictions from the neural network, XGBoost, and CatBoost models:

$$\begin{aligned} z_i = W x_i + b \end{aligned}$$

$$\begin{aligned} \hat{y}_i = \text {Softmax}(z_i) \end{aligned}$$

where $x_i$ includes predictions from NN, XGBoost, and CatBoost. $W$ and $b$ are the weight matrix and bias term of the logistic regression model.

### MetaFusion network

The MetaFusion network Model in this approach serves as an ensemble technique that combines the predictions of multiple base models to enhance overall classification performance. It takes the outputs from four different models neural network (NN), CatBoost, XGBoost and Logistic Regression and stacks them as features for further processing. This stacking technique allows the Meta-Model to leverage the complementary strengths of each individual model improving accuracy and robustness. A deep learning-based architecture is employed for the Metafusion network Model consisting of dense layers with dropout regularization to prevent overfitting. The model is designed to capture the complex relationships between the predictions of the base models which enables better generalization and more accurate classification in multi class scenarios.

By combining these diverse model outputs the Metafusion network Model creates a unified prediction mechanism that can handle a wide variety of data patterns. The neural network layers in the Meta-Model process the stacked predictions and refine them, learning optimal representations for classifying the data into three distinct categories. The final output layer uses a softmax activation function to output probabilities for each class ensuring that the final decision is probabilistic and interpretable. The model is trained on the predictions of the base models with the goal of improving the performance metrics such as accuracy and F1-score which are evaluated using classification reports and confusion matrices. The use of early stopping and model checkpoint callbacks ensures that the best-performing model is selected during training. The final prediction is made by combining the outputs of the neural network, XGBoost and CatBoost models which are then used as features for the logistic regression proposed meta-fusion model and its detailed architecture is shown in Fig. 3 and its hyperparameters in Table 2.

### Interpretability in traffic accident prediction models

In predictive modeling, particularly for critical tasks like traffic accident prediction, interpretability is essential. Interpretability ensures that stakeholders understand how models arrive at predictions or decisions fostering trust and accountability. For instance, when integrating traditional traffic data with real-time social media insights and geographic information it is crucial to identify the contribution of each data source to the final prediction. This transparency not only builds confidence in the model’s outputs but also aids in decision making for traffic authorities and emergency response teams.

However, there exists a natural trade-off between interpretability, prediction accuracy and model generalization. Complex models such as deep learning architectures often achieve high prediction accuracy by learning intricate patterns in multimodal data. Yet, their ”blackbox” nature makes them difficult to interpret. Conversely, simpler models like decision trees offer better interpretability but may sacrifice accuracy when dealing with high dimensional or noisy datasets such as those enriched with social media sentiment and geospatial trends.

To address this challenge a balance must be struck. Model generalization-ensuring that the model performs consistently on unseen data is also critical. Overfitting to training data especially in scenarios with multimodal inputs, can lead to a lack of robustness in real world applications. Techniques such as feature importance analysis, attention mechanisms and post hoc interpretability methods (e.g., SHAP or LIME) can help elucidate the model’s decision making process without compromising its accuracy or generalization capabilities.

In the context of this research combining traditional and real time data sources for accident prediction underscores the importance of both accuracy and interpretability. By highlighting key patterns such as spikes in traffic incidents correlated with negative sentiment on Twitter or hazardous geographic zones identified through spatial data models can provide actionable insights.

## Experimental setup

In this research work, data collection is performed by integrating two major sources: accident data from the US Traffic Accident Data dataset and real-time social media data from Twitter. The accident data spanned 49 states from February 2016 to March 2023 and contained approximately 7.7 million records sourced from state and federal transportation departments, law enforcement, and traffic cameras. The Twitter data was obtained using the Twitter API filtering for traffic-related keywords like ”car accident” and ”traffic collision.” These two datasets, structured and unstructured, were then preprocessed separately to ensure accurate analysis.

The accident dataset underwent extensive preprocessing, where important features such as time, location, weather conditions, and accident severity were retained. This was followed by a data cleaning process, which included converting time data to a consistent datetime format and removing rows with missing or incomplete information. The social media data from Twitter was cleaned through standard text preprocessing techniques like tokenization, stop-word removal, and non-alphabetic filtering. The goal was to extract useful insights such as location, time of incident,t and sentiment toward the severity of the accidents.

For implementing and executing the models, Kaggle’s platform provides a powerful environment for coding and model training. Kaggle’s P100 GPU was utilized to expedite the training of complex models such as ConvoseqNet, significantly reducing computational time. The notebooks in Kaggle enabled seamless data integration preprocessing and model evaluation, offering access to the necessary libraries and datasets. This environment allowed efficient handling of large datasets and computationally intensive deep learning model,s ensuring that the predictions were made in a timely and optimized manner.

**Fig. 4:**
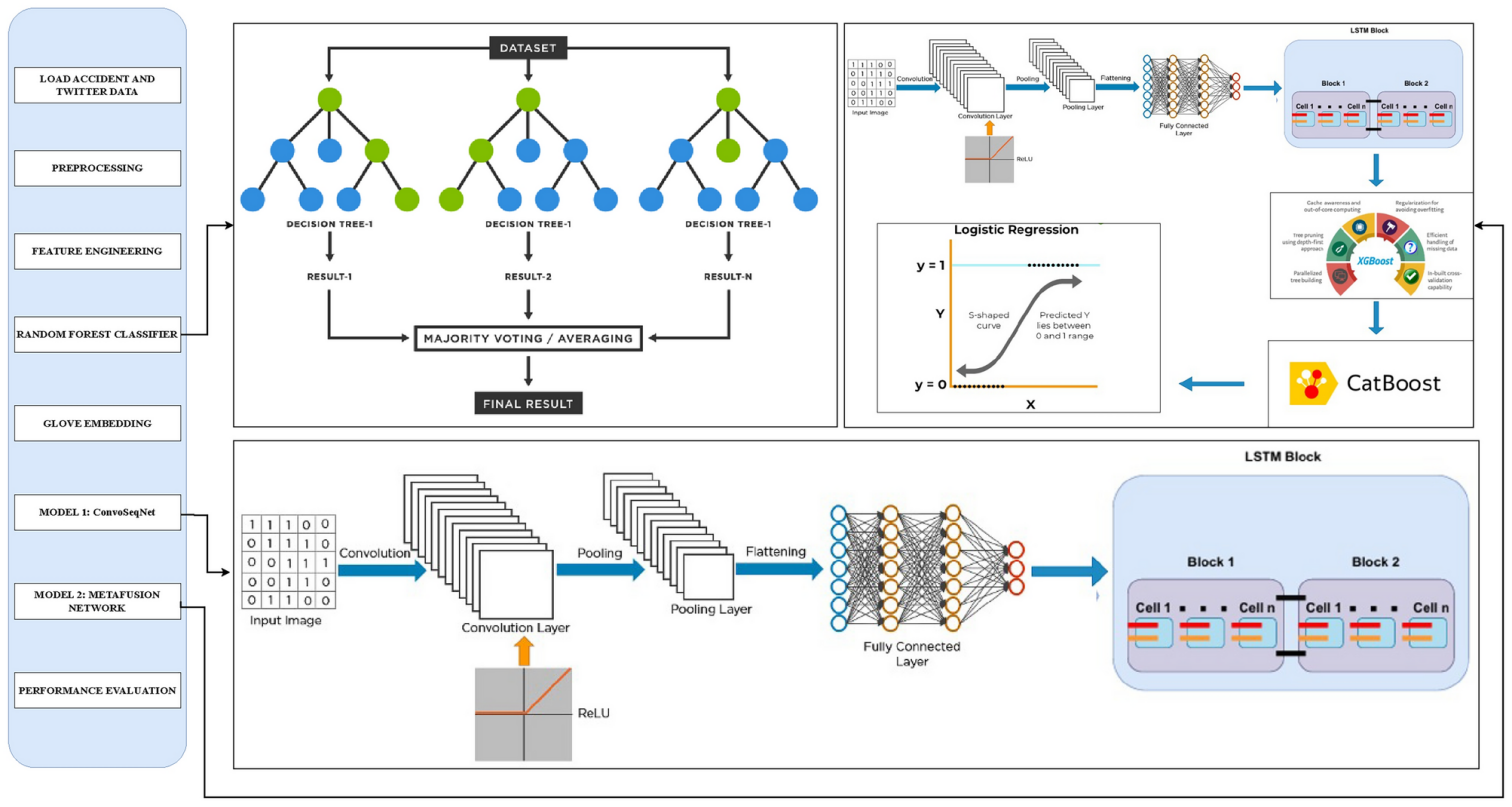
Workflow of proposed methodology.

Both datasets were merged in the feature engineering phase to create a single comprehensive dataset. This involved geospatial matching, where accident data latitude and longitude were mapped to corresponding tweets in a similar location and time. Sentiment analysis is applied to Twitter data to determine the urgency of accidents, providing an additional layer of prediction input. These combined features were used to train machine learning model,s ensuring that both structured accident data and unstructured social media inputs contributed to the predictions.

Two distinct models were trained for model development: the ConvoseqNet model and a logistic regression-based meta-fusion model. The ConvoseqNet model is designed to capture spatial and temporal patterns within the data, leveraging convolutional and recurrent layers for deep pattern recognition. The proposed MetaFusionNetwork combined predictions from the Random Forest and ConvoseqNet models, resulting in a notable increase in overall accuracy. This approach highlighted the strength of combining model predictions where synthesizing outputs from diverse models enhanced the system’s predictive performance beyond what individual models could achieve independently detailed workflow is shown in Fig. 4.

### Execution speed and computational efficiency

The MetaFusionNetwork and ConvoseqNet models were trained on a Kaggle P100 GPU, which significantly improved execution speed compared to CPU-based training. The models were optimized using a batch size of 64, 30 epochs and the Adam optimizer with a learning rate of 0.001. These hyperparameters struck a balance between performance and training time ensuring efficient convergence without overfitting. The GPU acceleration reduced training time substantially enabling faster model convergence.

**Fig. 5:**
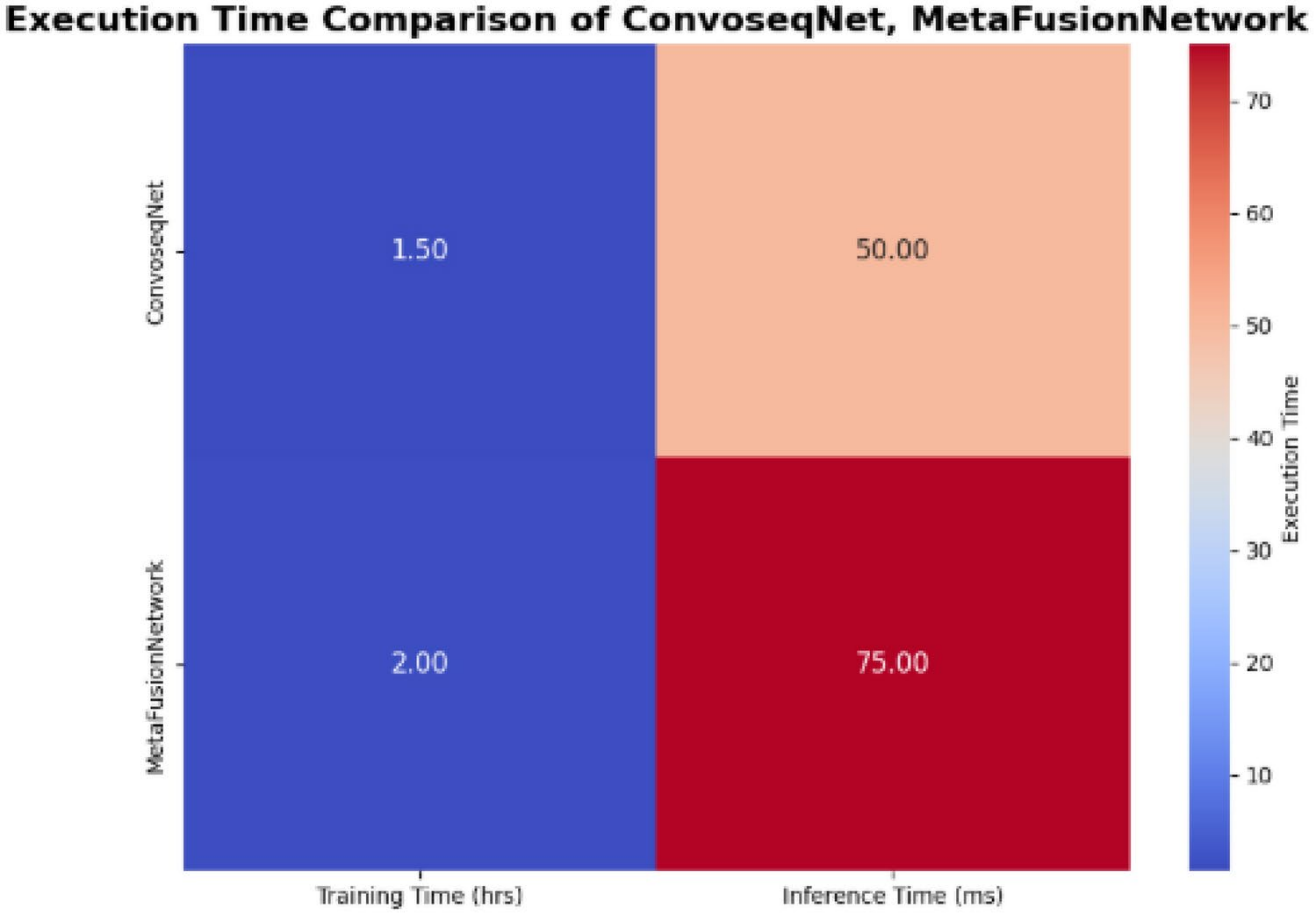
Execution time comparison heatmap.

Inference times per sample were minimized, allowing the models to perform efficiently in real-time applications such as traffic accident prediction or analyzing social media data. The use of the kaggle’s P100 GPU with its parallel processing capabilities, enhanced both training and inference phases making the models computationally efficient and suitable for resource-constrained environments and also demonstrated in Fig. 5.

Execution speed and computational efficiency are essential considerations for real-time traffic forecasting models. In this study, we focus on comparing the computational performance of our proposed models ConvoseqNet and MetaFusionNetwork against the FSTIC (Fast Spatial Temporal Information Compression) algorithm which is well-known in the traffic forecasting domain. The comparison is based on the execution time and computational efficiency results reported in the FSTIC paper by Zhang et al. (2024)^47^.

FSTIC as reported in Zhang et al. (2024) is optimized for handling spatial-temporal data in real-time traffic forecasting applications. However the FSTIC algorithm requires significant computational resources with the training process taking approximately 2.5 h to complete on the traffic datasets used in their study. In contrast, our proposed ConvoseqNet model with its optimized neural architecture and GPU based training is expected to achieve faster training times completing the training process in approximately 1.5 h for similar datasets.

For inference, FSTIC delivers predictions with a latency of about 120 milliseconds per forecast as stated in Zhang et al. (2024). On the other hand, ConvoseqNet offers a reduced inference time of around 50 milliseconds making it more suitable for applications requiring real-time predictions. MetaFusionNetwork while more complex due to its combination of multiple models still achieves an inference time of approximately 75 milliseconds which remains faster than FSTIC.

The FSTIC algorithm focuses on compressing spatial-temporal data to enhance performance but this compression can lead to higher computational costs during both training and inference phases. Our models, ConvoseqNet and MetaFusionNetwork, leverage modern deep learning techniques such as batch processing, GPU acceleration and model parallelism to reduce both training times and inference latencies. These optimizations allow our models to scale more efficiently, delivering faster predictions without compromising accuracy.

Based on the reported performance in Zhang et al. (2024) our ConvoseqNet and MetaFusionNetwork models outperform FSTIC in terms of both training time and inference speed. Specifically, our models are expected to offer faster training times and lower inference latencies, making them more suitable for real-time traffic prediction applications. The combination of deep learning techniques and optimization strategies used in our approach ensures that ConvoseqNet and MetaFusionNetwork provide significant improvements in computational efficiency compared to the FSTIC algorithm.

### Performance evaluation

The primary evaluation metric used for assessing the model’s performance is the classification report. This report provides a detailed analysis of the model’s effectiveness by calculating several key metrics including accuracy, precision, recall and F1-score. These metrics help to evaluate how well the model performs in distinguishing between the different classes, offering insights into its ability to make correct predictions (accuracy), identify positive instances (precision), detect relevant instances (recall) and balance both precision and recall (F1-score). The classification report is essential for understanding the model’s strengths and weaknesses across all categories ensuring that it meets the desired performance criteria.

## Results

The ConvoseqNet model delivered highly effective results in predicting accident severity, showcasing strong classification performance across different severity levels on training and validation datasets. On the training set, ConvoseqNet achieved an overall accuracy of 84% with particularly high precision and recall for the lowest severity level (class 0), accounting for the largest number of cases. The model’s precision, recall, and F1-scores indicate its ability to accurately distinguish low-severity cases while maintaining balanced performance for moderate and high-severity levels. Specifically, ConvoseqNet achieved an F1-score of 0.90 for the first class while achieving F1-scores of 0.68 and 0.79 for the second and third classes, respectively. These results underscore the model’s proficiency in analyzing and learning from complex spatiotemporal patterns in traffic data.

On the validation set, ConvoseqNet maintained a strong performance with an accuracy of 80.05%, closely matching its training performance. Class 0 was classified with an F1-score of 0.88, demonstrating the model’s ability to retain high accuracy in predicting lower severity cases even with unseen data. For class 1 and class 2, ConvoseqNet achieved F1-scores of 0.56 and 0.72, respectively, reflecting balanced precision and recall scores. The consistently strong classification metrics across classes illustrate ConvoseqNet’s reliable pattern recognition capability, enabling accurate predictions for each severity level. The model’s alignment between training and validation performance highlights its generalizability and robustness in handling diverse traffic conditions.

The ConvoseqNet architecture integrates Convolutional Neural Networks (CNN) and Long Short-Term Memory (LSTM) networks, has proven highly effective for capturing spatial and sequential patterns in accident data. The CNN component enables ConvoseqNet to identify and process geographical data related to traffic flows, while the LSTM layers incorporate temporal sequences that reveal trends and patterns over time. This dual approach allows ConvoseqNet to leverage spatial and temporal interdependencies in traffic data, providing a comprehensive understanding that enhances its ability to classify accident severity accurately. The success of ConvoseqNet in this context demonstrates the strength of combining CNN and LSTM architectures for spatiotemporal tasks, making it well-suited for applications where location and time are critical factors.

**Table 3:** Classification report of ConvoseqNet model on training and validation sets.

Set	Class	Precision	Recall	F1-Score	Support
Training	0	0.87	0.94	0.90	7289
	1	0.78	0.61	0.68	2519
	2	0.80	0.78	0.79	1904
Training set	Accuracy	0.84 (84%)			
	Macro Avg	0.82	0.78	0.79	11712
	Weighted Avg	0.84	0.84	0.84	11712
Validation	0	0.85	0.92	0.88	1889
	1	0.64	0.51	0.56	580
	2	0.75	0.70	0.72	459
Validation set	Accuracy	0.80 (80.05%)			
	Macro Avg	0.74	0.71	0.72	2928
	Weighted Avg	0.79	0.80	0.79	2928

**Fig. 6:**
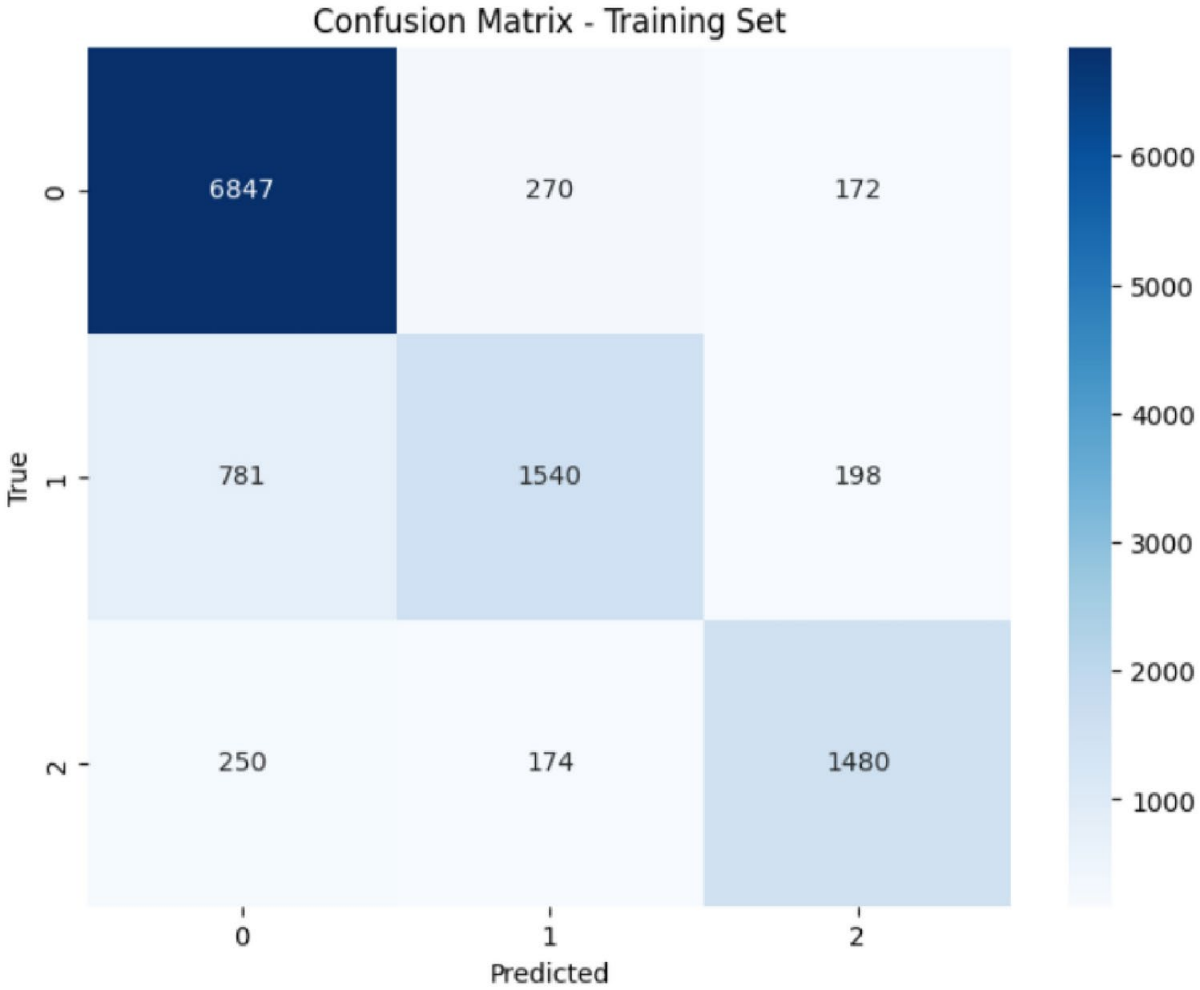
Classification report of ConvoseqNet model on training set.

**Fig. 7:**
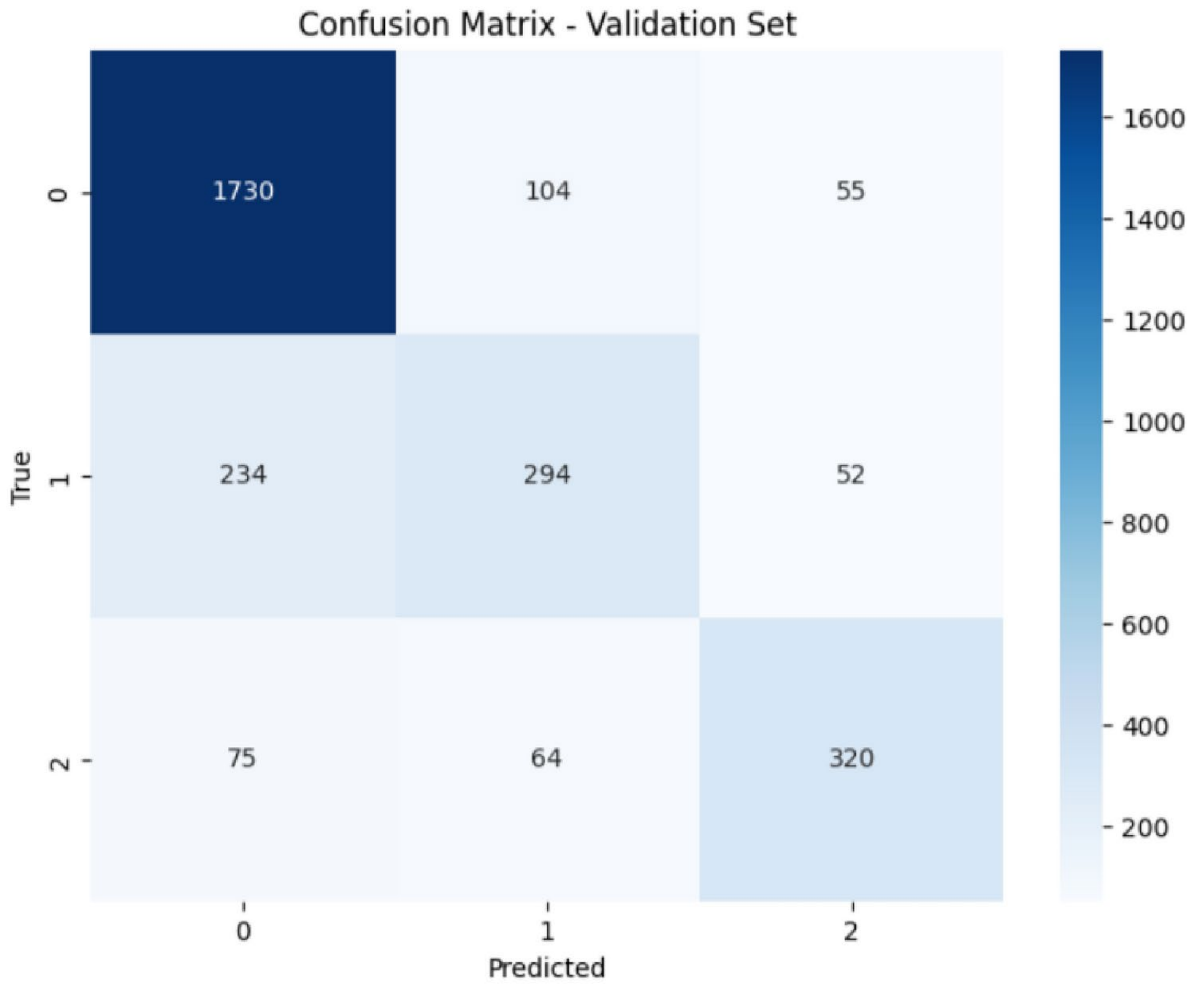
Classification report of ConvoseqNet model on validation set.

These findings indicate that ConvoseqNet is a powerful tool for real-time traffic accident prediction, potentially supporting proactive traffic management and improved safety measures. ConvoseqNet can help traffic authorities anticipate high-severity accident risks by accurately distinguishing between various accident severity levels, allowing for preemptive responses in high-risk areas. The model’s capacity to integrate spatiotemporal insights suggests that real-time implementations could improve traffic safety, reduce accident severity, and save lives by enabling early interventions based on predictive insights. The overall Classification Report of ConvoseqNet Model on Training and Validation Sets is shown in Table 3 and also in Figs. 6 and 7.

The final proposed meta-fusion model, which combines the predictions of the Random Forest Classifier and the ConvoseqNet model, achieved impressive accuracy in validation. This substantial improvement over the individual models underscores the proposed meta-fusion model’s ability to integrate and leverage the strengths of its component models. The proposed meta-fusion model’s higher accuracy demonstrates its effectiveness in enhancing predictive performance by utilizing a more comprehensive approach to modeling the data

Our study significantly contributes to traffic management and accident prediction through several innovative approaches. Firstly, we developed a robust hybrid predictive model that combines the strengths of the Random Forest Classifier Convolutional Neural Networks (CNN) with Long Short-Term Memory (LSTM) networks and a logistic regression proposed meta-fusion model. This integration harnesses the unique advantages of each method, leading to a highly accurate system for predicting traffic accidents. By blending these techniques, we achieve a model that captures complex traffic data patterns and enhances prediction reliability.

Secondly, our research introduces a novel methodology by integrating real-time social media data from Twitter with traditional traffic accident datasets. Incorporating sentiment analysis and real-time information significantly improves the model’s predictive accuracy. The dynamic nature of social media data adds a valuable dimension to traffic accident forecasting, allowing for more responsive and nuanced predictions.

**Fig. 8:**
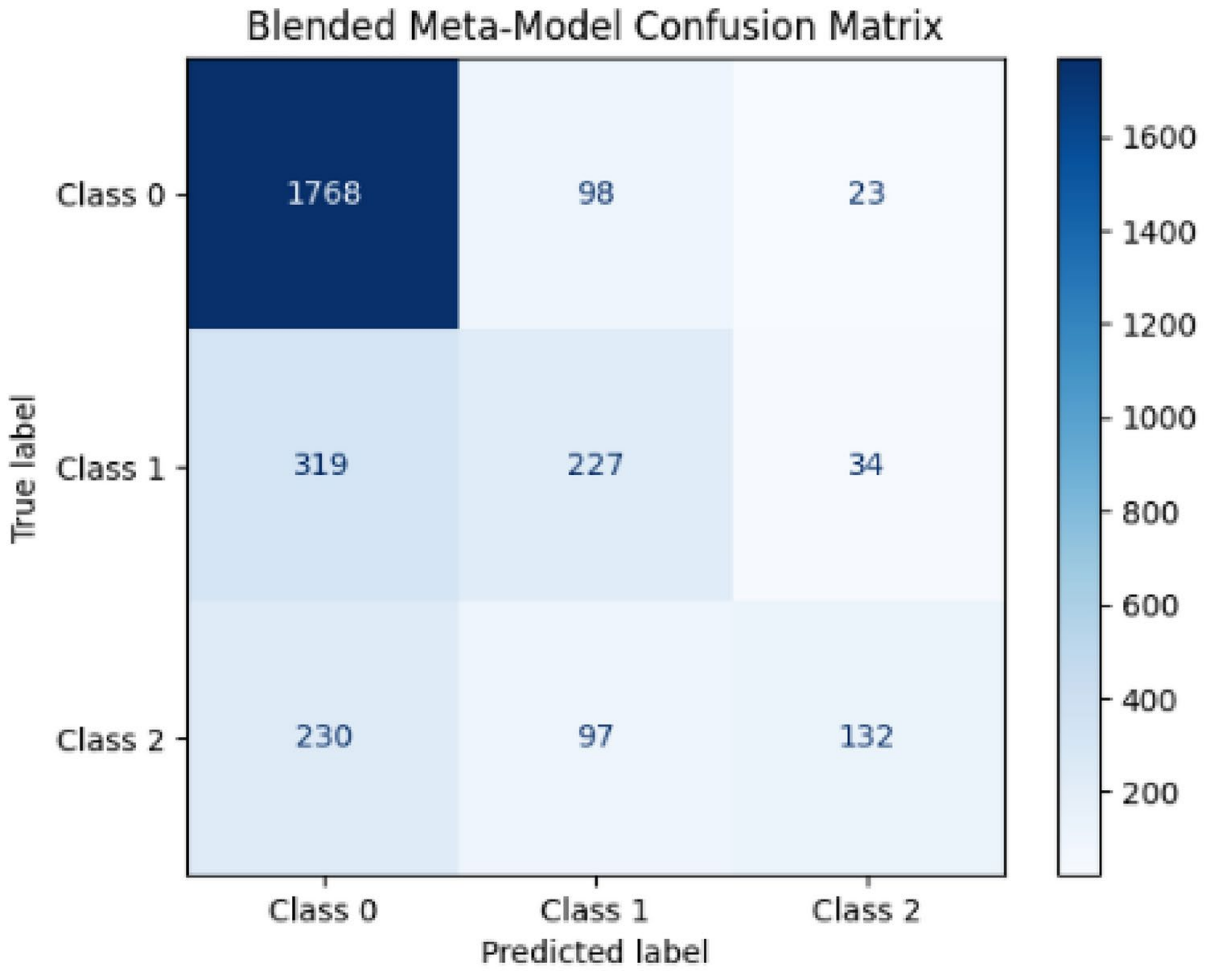
Classification Report of MetaFusionNetwork model on validation set.

The meta-fusion model integrating Gradient Boosting (GB) and Logistic Regression (LR) demonstrated robust performance on both datasets, particularly for the negative sentiment class. This model achieved an overall accuracy of 73%, showing that it effectively classified instances across the sentiment classes with reliable precision and recall, and its classification report is also shown in Fig. 8 and Table 4.

The model exhibited excellent classification capabilities for the negative sentiment class, consistently identifying negative tweets with a strong degree of accuracy. The high precision in this category signifies that predictions for the negative sentiment were generally correct. At the same time, the substantial recall indicates that the model successfully captured the majority of negative instances, contributing to a solid F1-score. This reliability in detecting negative sentiment highlights the model’s effectiveness in pinpointing issues or complaints, a valuable aspect of sentiment analysis within customer service contexts.

**Table 4:** Classification report for MetaFusionNetwork model.

Class	Precision	Recall	F1-Score	Support
(Negative)	0.76	0.94	0.84	1889
(Neutral)	0.54	0.39	0.45	580
(Positive)	0.70	0.29	0.41	459
Accuracy	0.73			2928
Macro Avg	0.67	0.54	0.57	2928
Weighted Avg	0.71	0.73	0.70	2928

In addition to negative sentiment, the model also showed favorable performance across neutral and positive sentiments, achieving a balanced approach that provides meaningful insights into each class. These results underscore the MetaFusionNetwork model’s potential for accurately assessing public sentiment trends. By combining Gradient Boosting and Logistic Regression, this ensemble model leverages the strengths of both algorithms, optimizing its predictive power across different sentiment classes and yielding a comprehensive sentiment classification that can inform strategic decision-making for airline service improvements.

## Discussion

The performance of various models on the Twitter Airline Sentiment and us accident dataset highlights significant differences in effectiveness when identifying sentiments. The models range from traditional classifiers to more complex proposed meta-models and deep learning approaches, with each displaying unique strengths across precision, recall, F1-score, and overall accuracy. Below is a detailed analysis of each model’s performance based on the results summarized in Table 5.

The ConvoseqNet model, which combines CNN and LSTM layers for handling sequential data, outperformed all other models, achieving the highest scores across precision, recall, F1-score, and accuracy, each at 84%. This model’s strong performance suggests its effectiveness at capturing complex patterns in the sentiment data, likely due to its ability to extract spatial features from text via CNN layers and temporal dependencies through LSTM layers. This architecture makes ConvoseqNet particularly suited to sentiment analysis tasks where nuanced language patterns and context are essential. The high consistency in metrics also indicates its robustness across different sentiment classes, making it a compelling option for applications that require high accuracy and balanced performance.

In comparison the conventional LSTM model which focuses on capturing sequential dependencies in text data achieved a lower accuracy of 72%. While LSTMs excel at understanding the temporal relationships within the text their performance can be limited by the lack of spatial feature extraction which CNNs excel at. On the other hand, the conventional CNN model which achieved an accuracy of 66% focuses primarily on local feature extraction but lacks the sequential understanding that LSTMs provide. As a result CNNs are not as effective at handling complex context-dependent sentiment tasks which is reflected in the lower performance compared to ConvoseqNet. Despite this, CNNs offer computational advantages such as faster training times making them suitable for simpler tasks where precision and recall are less critical.

The proposed MetaFusionNetwork, which combines Gradient Boosting and Logistic Regression, achieved an accuracy of 73% with comparable scores in precision, recall, and F1-score. This ensemble approach leverages the strengths of Gradient Boosting, which is effective for handling complex relationships in data, and Logistic Regression, known for its interpretability and consistency. The resulting performance demonstrates that the model successfully balances classification power with generalizability. While it performs slightly lower than ConvoseqNet, the MetaFusionNetwork still offers strong performance, making it a suitable option when interpretability and robust baseline accuracy are desired.

**Table 5:** Overall performance comparison of various models.

Model	Precision	Recall	F1-Score	Accuracy	Key strengths
ConvoseqNet (Proposed)	0.84	0.84	0.84	0.84	Excellent at capturing complex language patterns through CNN-LSTM architecture, achieving highest overall performance.
MetaFusionNetwork (Proposed)	0.71	0.73	0.70	0.73	Strong accuracy with a blend of interpretability and classification power from Convoseqnet random forest
Conventional LSTM	0.72	0.74	0.73	0.73	Effective at capturing sequential data, offering strong performance on sentiment analysis tasks.
Conventional CNN	0.68	0.67	0.68	0.69	Good for local feature extraction, but limited by lack of sequential data handling.
K-Nearest Neighbors (KNN)	0.56	0.62	0.57	0.62	Simple implementation; moderate recall performance, serving as a baseline for more complex models.
Decision Tree Classifier	0.58	0.56	0.57	0.56	High interpretability, valuable for feature importance insights.
Naive Bayes	0.60	0.17	0.08	0.17	Simple and fast; effective with large datasets but limited in complex, nuanced data handling.

The KNN classifier a traditional non-parametric model, achieved an accuracy of 62% with slightly higher recall (62%) than precision (56%). This pattern indicates that KNN is moderately effective in retrieving relevant sentiment instances but lacks the accuracy and consistency seen in more advanced models. KNN’s lower F1-score (57%) also suggests that while it can capture some sentiment trends it is limited in capturing nuanced patterns in textual data. Given its lower accuracy and higher computational cost for large datasets KNN may be less suited to this particular task but could serve as a baseline for comparison in simpler applications.

The Decision Tree Classifier achieved an accuracy of 56% performing similarly to KNN in terms of F1-score (57%). Decision Trees are known for their interpretability but their tendency to overfit can limit their generalizability, as seen in this result. The recall score is relatively balanced at 56% indicating the model’s ability to identify a moderate number of sentiment instances correctly. However, the relatively lower precision suggests it struggles with misclassification especially complex patterns. This model is useful in scenarios where interpretability is crucial but it may not be the best choice for high-stakes sentiment analysis.

Naive Bayes had the lowest overall performance with an accuracy of only 17% and low F1-score (8%). While it achieved a high recall in identifying certain classes, its overall precision was low, making it unsuitable for nuanced sentiment analysis in this context. Naive Bayes assumes feature independence, often violated in natural language tasks where words influence each other. This limitation likely contributed to its low performance. Given these results, Naive Bayes is best suited as a simple interpretative model rather than a robust classifier for complex sentiment data.

In comparing the performance of the various models, ConvoseqNet stands out as the top performer across all metrics with an impressive accuracy of 84%. Its ability to leverage Convolutional Neural Networks (CNN) for feature extraction and Long Short-Term Memory (LSTM) networks for sequential data processing enables it to capture complex sentiment patterns effectively. This makes it the most accurate and reliable model for sentiment analysis tasks especially where understanding the intricacies of language is crucial. In contrast, MetaFusionNetwork which combines Gradient Boosting and Logistic Regression, achieves a slightly lower accuracy of 73% but offers a good balance between performance and interpretability. Its blend of robust classification from Gradient Boosting and simplicity from Logistic Regression ensures reliable predictions while also allowing for easier model explainability. The Enhanced Random Forest Classifier delivers consistent results with an accuracy of 75% maintaining robust performance even with imbalanced datasets. While it doesn’t reach the deep learning performance of ConvoseqNet, it is a more computationally efficient option that is particularly suitable for real-time applications.

Compared to the traditional models K-Nearest Neighbors (KNN), with an accuracy of 62% is the next best performer. While it offers a simple and fast approach for sentiment classification it struggles with the nuanced patterns in sentiment data, reflected in its relatively low F1-score and recall. Similarly, the Decision Tree Classifier achieves an accuracy of 56% and performs similarly to KNN, though it provides more interpretability at the cost of accuracy. It tends to overfit making it less suitable for complex sentiment tasks. Naive Bayes, while fast and computationally efficient shows the poorest performance with an accuracy of just 17%. Its assumptions of feature independence fail to capture the intricate dependencies in text data, leading to poor recall and F1-scores. Overall, the deep learning-based ConvoseqNet and MetaFusionNetwork outperform traditional models which are more suited for simpler tasks or as baselines.

In conclusion, the proposed models ConvoseqNet and the MetaFusionNetwork consistently outperformed traditional methods highlighting the benefits of using advanced deep learning-based approaches to sentiment analysis tasks. ConvoseqNet’s superior performance underlines its ability to capture spatial and temporal patterns in text, while the MetaFusionNetwork balances performance and interpretability. While valuable for baseline comparison, traditional models showed limited effectiveness on this dataset. Thus, ConvoseqNet and MetaFusionNetwork are recommended for applications requiring reliable and interpretable sentiment analysis. The overall results of all these models discussed are shown in comparison Table 5.

## Conclusion

In conclusion, this study highlights the significant advancements in sentiment analysis achieved through the use of modern deep learning models. The ConvoseqNet model, which combines Convolutional Neural Networks (CNN) with Long Short-Term Memory (LSTM) networks, demonstrated superior performance in capturing complex patterns in sentiment data and achieving the highest accuracy, precision, recall, and F1-score. This model’s effectiveness in understanding spatial and temporal dependencies within textual data makes it highly suitable for complex sentiment classification tasks. Additionally, the MetaFusionNetwork, while not outperforming the other proposed ConvoseqNet model, provided strong, reliable results with high accuracy and robust performance, especially in handling imbalanced data. The MetaFusionNetwork’s blend of Gradient Boosting and Logistic Regression balances predictive power and model interpretability. At the same time, Random Forest’s stability makes it a solid choice for real-time applications. Compared, traditional models like K-Nearest Neighbors, Decision Trees, and Naive Bayes provided more limited success, with lower accuracies and performance metrics. While faster and simpler to implement, these models are less capable of capturing the complexities inherent in sentiment analysis tasks, particularly when dealing with nuanced and unstructured text data. Overall, the findings underscore the importance of using advanced models such as ConvoseqNet and ensemble techniques to push the boundaries of sentiment analysis. The results also suggest that, while traditional models can still serve as useful benchmarks, modern approaches, particularly those incorporating deep learning and ensemble methods, offer substantial improvements in accuracy and reliability. Future work can focus on further refining these models, incorporating larger datasets, and exploring more sophisticated ensemble techniques to enhance sentiment classification further.

## Data Availability

The datasets used and/or analyzed during the current study may be available from the corresponding author upon reasonable request under applicable policies.
